# Discolation of the Eye-ball in Dogs

**Published:** 1887-07

**Authors:** Daniel D. Lee

**Affiliations:** Instructor in Anatomy, and Operative Vetinary Surgery, Harvard University


### ART XXV.—DISLOCATION OF THE EYE-BALL IN DOGS.

BY DANIEL D. LEE, M. D. V.

*Instructor in Anatomy, and Operative Vetinary Surgery, Harvard University.*

This is of rather common occurence in dogs with prominent eyes, and is generally the result of a fight; I have seen one case which was caused by the dog being struck by a locomotive; no other injury was sustained, and the eye was entirely unhurt except for the dislocation.

On this subject Williams says in his work on Veterinary Surgery: "The method of returning the eye is as follows: After washing away all extraneous matter, dirt, blood, etc., with tepid water, let an assistant, who is to stand behind the dog, open the eyelids as far apart as possible, then the operator is to press gently but firmly upon each side of the anterior aspect of the dislocated globe, with the balls of the thumbs, until the globe is replaced within the orbit. But should such pressure prove ineffectual, the outer angle of the eyelid is to be divided with a pair of scissors,—when the eye can be replaced without difficulty." The incision in the lids closed, he further says that "*extreme pressure* must be avoided, or the eye will be irretrievably damaged;" this I do not think is so unless the eye has been *lacerated* as well as *dislocated.* The eye is found hanging well down on the face, held in place by the conjunctiva and muscles, the last having lost their tone by the extreme tension.

In my treatment I have found that the best way to replace the eye, and that attended with the most satisfactory results is to etherize the dog, carefully cleanse the eye with warm water, and clip away all hair about the parts. Turn the edges of the lids out with a probe, if they are turned in, clip off the eyelashes, and then make *extreme* and sudden pressure on the sides of the anterior part of the eye with the thumbs, following it up until the eye gives a slip back into the orbit; if successful, the eye will remain in the socket and *not* protrude when the thumbs are removed. Now run a probe between the lids and eyeball as far as the reflection of the conjunctiva (conjunctival sinus), bringing it around the eye. This smoothes and folds the mucous membrane into place. The membrana nictitans may protrude from the swelling of the parts and is sometimes permanently returned by the use of the probe, at others it comes out again, but generally retracts after the inflammation has subsided; if not it may be amputated. Having replaced the eye take a stitch in the outer third of the lids as a precautionary measure. Then hold a lump of ice over the parts for a few minutes, or bathe with very cold water; this contracts the parts and makes the muscles of the eye regain their tone, and thus holds the eyeball in place.

Tie the fore legs firmly together with a narrow roller bandage and place a large pad of absorbent cotton over the eye, bandaging it firmly on. Leave this bandage alone for two days; at the end of this time if the operation is to be a success, the stitch may be removed and the bandage left off. The following wash is very good

℞. Sodae Biboratis, gr. x.
Aquae Camp, oz. i.
*Sig.* Drop 10 m. in eye t. i. d.

If dislocation does *not* recur, a good prognosis may now be given, and if the eye was not lacerated the sight will in all probability be unimpaired. If dislocation returns when the bandage and stitch are removed—it seldom does if the eye was properly returned—it is generally necessary to amputate the cornea and evacuate the media of the eye.

In long haired dogs the stump thus formed is almost entirely hidden and is no disfigurement.

When reduction is not complete at the beginning and stitches are taken in the lids to hold the eye-ball in place, the ensuing swelling pushes the eye-ball against them and they cut the cornea, finally tearing out, allowing the dislocation to recur and making amputation at cornea, or of the entire globe necessary. If extreme pressure is used, I do not think it is ever necessary to cut the lids; if the leison is several hours old the parts may have become very dry and require oiling.

Says Lecoq: "It is only necessary in a fresh eye to squeeze the globe in order to produce a degree of opacity in the cornea which will be more or less great in proportion to the amount of pressure exercised." From this we need not be surprised to learn that the cornea is more or less clouded for a week or two after reduction by this method.

I will add that keeping the dog in a moderately dark room for two weeks is advised, and also that the bandage fastening the fore legs together should not be taken off for three or four days, or the scratching is almost sure to injure the eye.

It may appear that I have written at length on an unimportant subject, but as I have found that these cases are most gratifying when successful and exceedingly disagreeable when failures, I thought that my experience might be of interest.

